# Development of Defensive Actions in Small-Sided and Conditioned Games With Offensive Purposes in Futsal

**DOI:** 10.3389/fpsyg.2020.591572

**Published:** 2020-10-26

**Authors:** David Pizarro, Alba Práxedes, Bruno Travassos, Alberto Moreno

**Affiliations:** ^1^Faculty of Languages and Education, University of Nebrija, Madrid, Spain; ^2^Department of Sport Sciences, Research Center in Sport Sciences, Health and Human Development (CIDESD), University of Beira Interior, Covilhã, Portugal; ^3^Portugal Football School, Portuguese Football Federation, Oeiras, Portugal; ^4^Faculty of Sport Sciences, University of Extremadura, Cáceres, Spain

**Keywords:** futsal, defensive actions, task constraint, performance analysis, non-linear pedagogy

## Abstract

Based on ecological dynamics approach, non-linear pedagogy (NLP) have emerged with the goal of promoting a holistic approach through the use of small-sided and conditioned games (SSCGs), to optimize specific tactical defensive and offensive behaviors of players. This study analyzed the indirect effects of an intervention program, based on NLP (task design based on tactical principles of attack and numerical advantage of attacking team), in decision-making (DM) and execution (Ex) in defensive technical–tactical actions in U16 futsal. Eight futsal players (U16 years) participated in 12 training sessions, spread over two phases: preintervention and intervention. The Game Performance Evaluation Tool (GPET) instrument was used to analyze the DM and Ex of 2,600 defensive actions measured during competitive matches. Results showed significant improvements in marking actions (to the player with the ball: DM, *p* = 0.001; Ex, *p* = 0.001; and to the player without the ball: DM, *p* = 0.039; Ex, *p* = 0.046), improvements in blocking actions (DM, *p* = 0.015), and improvements in help-coverage actions (Ex, *p* = 0.014). No significant differences were found in the interception and tackling actions. This study has shown evidence that the NLP approach is an appropriate theoretical framework to enhance acquisition of defensive tactical behavior in futsal. However, not all actions improved. Therefore, coaches should design representative tasks to optimally develop technical–tactical training processes based on the phases of futsal game (offensive and defensive) and considering the level of opposition.

## Introduction

In team sports such as futsal, in which predominate open motor skills, it is required that players continuously coadapt their actions to the movements of opponents and teammates to ensure a functional collective behavior (Chow et al., 2016). It is a sport where tactical components assume a fundamental role in the effectiveness of each game action (López, 2017). Tactical behavior is a general concept that helps to explain how players guide behavior to functionally perform. From the perspective of ecological dynamics, tactical behavior is an active and continuous process of searching and exploration of relevant information to perform under the changes of the game (Gonçalves et al., 2014), which can be considered into two specific phases of game, according to game requirements: (a) with ball possession (offensive actions) and (b) without ball possession (defensive actions) (Corrêa et al., 2012). Despite the importance of achieving functional tactical behavior in both phases, it is generally considered that the development of defensive tactical behavior is usually considered of low priority when compared with offensive ones (Martiño, 2018). Indeed, previous research has ignored the analysis and development of defensive tactical behaviors (Reis et al., 2019), even being a key moment in the motor learning phase in team sports (Memmert and Roth, 2007).

López (2017), in a book from the European Union of Football Association (UEFA), referred that, to optimally develop the training processes of the defensive phase of futsal game, coaches should develop defensive tactical behavior of players by highlighting: (a) the perceptual demands associated with defensive actions [such as the identification of ball trajectory, position of teammates and opponents, orientation of the game (offensive orientation of the holder of the ball and collective defensive orientation based on the strong side–weak side), and the level of pressure on the ball (passing lines and direction of play of the offensive game)]; (b) the defensive foundations that support players’ defensive actions (man-marking, controlling the space, putting pressure on the ball, commanding the space, floating cover, switching opponent, closing/blocking passing angles…); and (c) the defensive game principles that support collective behavior (recover the possession of the ball, prevent progression, and avoid the goal (Bayer, 1992; Martiño, 2018), the strategic requirements that support collective behavior under game context [such as the type of defense used (individual or marking zone), the collective defensive moves according with the ball position on the field, or even the defensive tactical intention according to risk assessment (result, time.)] (see Table 1).

**TABLE 1:** Relations between technical–tactical actions with foundations that support players’ defensive actions and the defensive game principles.

**Defensive game principles**	**Defensive foundations**	**Technical-tactical actions**
Recover the possession of the ball	Obstruction of pass lines	Marking *(defensive action to player with the ball)*, tackling;
	Individual aspects of marking	marking *(defensive action to player without the ball)*,
	Pressure to the ball	interception and help coverage
Prevent progression	Defensive organization	Marking (*defensive action to player with the ball*), marking
	Timing	*(defensive action to player without the ball)*, interception
	Pressure to the ball	and help coverage
	Defensive deployment	Marking *(defensive action to player with the ball)*
Avoid the goal	Obstruction of shots	and blocking
	Individual aspects of marking	

*Self-made figure based on Contreras Jordán et al. (2001) and López (2017).*

Thus, to improve players’ tactical defensive behavior, it is required that the design of training tasks exposes players to game contexts that sample the perceptual-motor and strategic demands of competition according to the level of players (Travassos et al., 2012b; Xavier de Andrade, 2019). That is, training tasks and the consequent main goals should highlight perceptual, action, and strategic requirements of players’ actions according to players’ development and actual action capabilities.

In the last few decades, based on ecological dynamics approach, non-linear pedagogy (NLP) have emerged with the goal to promote a holistic approach through the use of small-sided and conditioned games (SSCGs) (Chow et al., 2016). The use of SSCG allows coaches to optimize specific tactical defensive and offensive behaviors of players by breaking the game in specific game subunits, i.e., GK + 1 × 1 + GK until GK + 3 × 3 + GK (Sampaio and Maçãs, 2012) instead of replicating the general demands play of real match (Aguiar et al., 2012; Sarmento et al., 2018). In line with this, coaches should go from simplified units with low number of players, to highlight the informational constraints that promote the development of defensive foundations of players, to more complex units until the numerical relation of the game to develop the game principles and strategic requirements that support collective behavior of teams according to the perceptual and action demands of competition. As such, modifying the number of players during the SSCG can lead to different opportunities for action.

Therefore, other types of constraints should be explored to assist coaches in the creation of more adjusted learning environments to their purposes (Santos et al., 2020). Different task constraints can be manipulated to boost the discovery and exploration of players’ movement solutions (Hristovski et al., 2011). To promote such motor exploration for adaptability, the available research has been suggesting the creation of tasks with additional variability (Seifert et al., 2013). In line with this, functional variability, as a principle emanating from an ecological dynamic (Chow, 2013), encourages the emergence of functional movement solutions within game situations (Schmidt and Lee, 2019) through improvement of perceptual attunement (i.e., capability to identify and explore the better information for performance in each context of practice) and motor calibration (i.e., capability to adjust movement solutions to spatial–temporal conditions of game) to game environments (Seifert et al., 2013; Pizarro et al., 2019).

In this perspective, the teaching process should be focused on the manipulation of relevant task constraints that highlight the informational constraints that support and guide players toward the functional resolution of each task goals according to the coaches’ main purposes (Passos et al., 2008). This is particularly relevant since they change the way players explore and act on the game context (Passos et al., 2008). In this line, it seems to indicate the different scenarios leading to the development of different capabilities through the emergence of different adaptive actions (Gonçalves et al., 2016). Therefore, to accomplish the main purpose of the development of, for instance, defensive players’ behavior, it assumes a major relevance of knowing which task constraints guide players to better exploit defensive individual fundamentals and actions or collective behavior according to their age or skill level (Davids et al., 2013; Gonçalves et al., 2016).

Previous studies clearly revealed how the modifications of task constraints can change players’ behavior (Travassos et al., 2014a; Vilar et al., 2014; Castellano et al., 2016; Gonçalves et al., 2017; Ometto et al., 2018). Curiously, task constraints as task aim (specifically the accomplishment of game principle to perform), balance on the number of outfield players, or instructional strategies as questioning are recently being studied (Sampaio et al., 2014; Travassos et al., 2014b; Serra-Olivares et al., 2015; Pizarro et al., 2019).

However, studies that analyze actions based on defensive game principles are not found in the scientific literature. In this sense, although the NLP approach, and more specifically the modified games, works in a holistic way the phases of attack and defense, if we design training programs focused on tactical principles of attack and with offensive numerical superiority, will the defensive technical–tactical actions improve?

Therefore, the main objective of this study was to analyze the indirect effects on defensive tactical behavior caused by the application of an intervention program based on attack, from the perspective of NLP (task design based on tactical principles of attack and offensive numerical advantage), on different defensive technical–tactical actions futsal.

## Materials and Methods

### Participants

The participants were eight male futsal players from the Under-16 category from two Spanish clubs (natural group not modified for research) as that of Pizarro et al. (2019). All had the same age (*M* = 15.375 and *SD* = 0.517) and level of expertise (i.e., average skill level) and participated in a regional league. In addition, players had sport expertise in futsal (*M* = 4.875, *SD* = 3.313).

The research project was fully approved by the Ethics Research Committee of a Spanish University. The research was developed under the recommendations of the Declaration of Helsinki, with respect to participant assent, parent/guardian consent, confidentiality, and anonymity. The participants and their parents were informed of the study, and an informed written consent was obtained from the parents/guardians.

### Design and Procedures

The study design consisted of an intragroup design, from a quasi-experimental methodology, where two research phases were considered. In the first phase, named preintervention phase, the values of decision-making and execution were recorded for the defensive actions analyzed [decision-making (DM) and execution (Ex)] in three competition matches, as indicated by previous studies (Práxedes et al., 2018b). This first measurement allows to establish the previous level of the players. In the second mentioned phase, i.e., intervention phase, the intervention program was developed. This program had a duration of 12 training sessions (minimum recommended by studies such as Harvey et al., 2010). As in the previous phase, the variables analyzed (DM and Ex) were recorded in competitive matches.

### Intervention

In preparation for the intervention, several meetings were conducted between the coach and the main research with the following goals: (a) discussion of NLP approach, (b) definition of discussion practice task contents, (c) design of tasks based on the principles of NLP, and (d) test of the tasks designed in a futsal team of the same age category as the participants of the present study (Harvey et al., 2010; Práxedes et al., 2016).

The design was conducted in 12 training sessions (≥ 12, as recommended by Harvey et al., 2010), 2 weekly sessions, for 6 weeks. In each training session, there were four learning tasks (without an active recovery between them) each lasting a total of 15 min, which was based on the use and manipulation of SSCG (see the example in Table 2). Extra balls were placed around the field to allow a quick restart of the task in case the ball went out of bounds. It is important to point out that there was no warmup because the intensity of the tasks was increasing.

**TABLE 2:** Example of a training session.

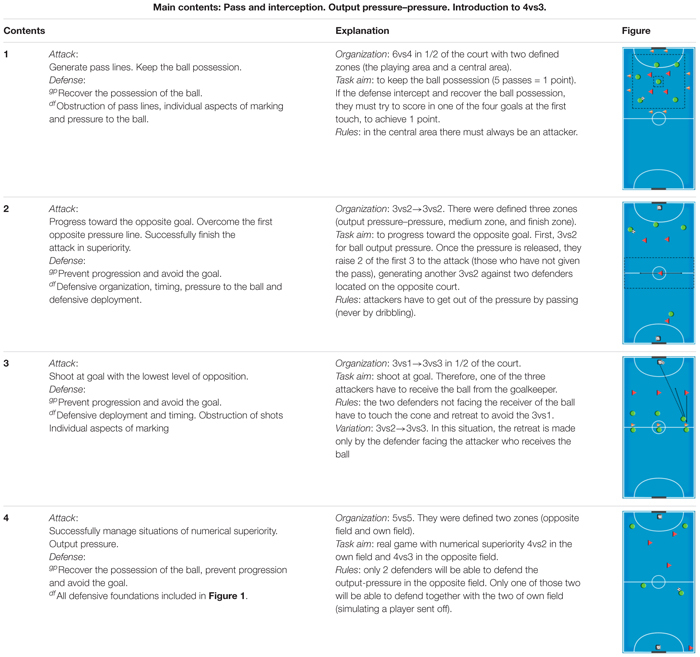			

*^*gp*^game principles; ^*df*^defensive foundations.*

More specifically, each modified game focused on the development of one of the offensive tactical game principles (Bayer, 1992). In this sense, the aim of the first task was to keep the ball possession; the aim of the second task was to progress toward the opposite goal, and that of the third was destined to the development of the principle to shoot at goal with the lowest level of opposition. No coach feedback or encouragement was allowed during the first (7 min) and the second (7 min) part of each task. Questioning was only implemented in the middle part of each task (1 min), from an ecological perspective (questions thrown by the coach in order to guide the players toward the objectives of the task without a conversation between them).

On the other hand, the level of opposition (level of numerical unbalance) was considered (Sampaio et al., 2014; Práxedes et al., 2018a). In all the tasks considered, a numerical superiority in attack was observed to highlight different offensive principles. Even not being the main goal of each SSCG, concurrently defensive foundations and tactical principles of play were also present in each task, allowing defensive players to also develop their defensive tactical capabilities of play.

**FIGURE 1:**
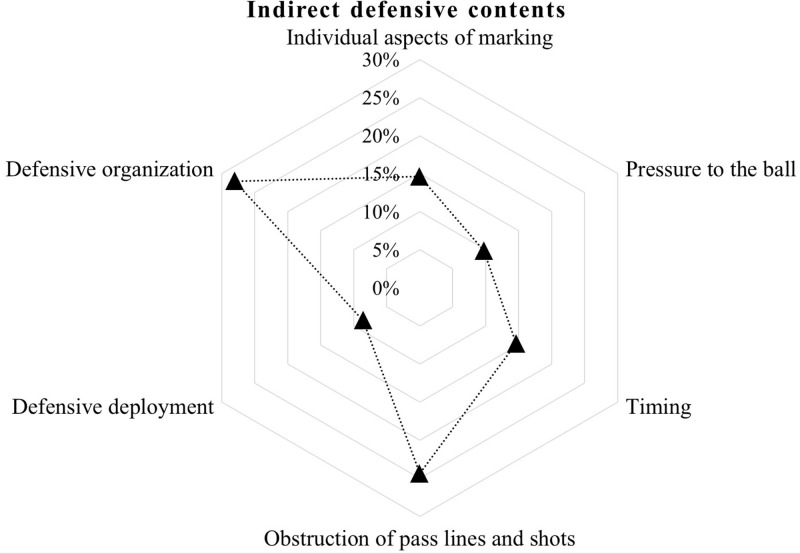
Percentages of indirect work for each of the defensive foundations included as contents in training session. Self-made figure based on López (2017).

The percentage of work of each defensive content was calculated for each exercise (see Figure 1). Each content in each task was registered as “1.” At the end of all sessions, the sum of each content was divided by the sum of total contents of sessions (in our case 82) to calculate the percentage of work of each content; for example, “individual aspects of marking:” 12/82 = 15%).

### Data Collection

The tactical defensive behavior of players was analyzed by considering DM and Ex of player’s behavior. To assess the DM and Ex of players, the Game Performance Evaluation Tool (GPET) observation instrument was used. This instrument permitted evaluating the player’s tactical problem-solving skills, by means of selecting an appropriate technique, and evaluating the measure in real-game situations, as recommended by Travassos et al. (2013).

*Decision-making*, as the process whereby athletes select one type of attack from a series of alternatives to execute it at a specific moment and in a real-game situation (Bar-Eli et al., 2011), was measured as the percentage of successful decisions over the total number of decisions made (García-López et al., 2013). The decision-making assessment was based on indirect and external systematic observation, a methodology that had been used in previous studies to measure players’ decision-making in real-game situations, which represents the influence of the environment on decision-making (Travassos et al., 2013). Through this instrument, decision-making was coded as 1 if successful (e.g., for marking action, defender tries to maintain the placement between the ball and the goal defended) or 0 if unsuccessful (e.g., for blocking action, defender does not try to block a shot) (García-López et al., 2013).

*Execution*, as the performance, outcome, or the final result of the motor execution (Bar-Eli et al., 2011), was measured by the percentage of successful execution over the total number of executions made (García-López et al., 2013). Through this instrument, execution was coded as 1 if successful [e.g., for tackling action, defender tries to recover the ball when he has an advantage (bad control or unsafe dribbling by the attacker player)] or 0 if unsuccessful (e.g., for marking action to the player without the ball, the marked attacker gets an advantageous position) (García-López et al., 2013).

The technical–tactical actions were analyzed considering if the defender marked the player with the ball (marking, blocking, and tackling) or the attacking players without the ball (marking, interception, and help coverage). Marking the player with the ball is defined as an action in which the defender will be placed between the opponent, the ball and the goal; interception is understood as the moment in which a defender stops an offensive action of the opponent throwing himself directly to the ball; tackling as a technical action allows the defender to conquer or move away the ball that is in the possession of an opponent; marking the player without the ball is an action in which the defender must always be placed between the opponent and the goal, inside the triangle formed by the position of the ball, that of the attacker, and the center of the goal; blocking is an action done by placing the body itself between the attacker who throws the ball and the goal that is defended, along a straight line that joins the direct rival with the goal itself; and help coverage is an action depending on the position of the attacker in relation to the ball and the goal, that is when the attacker is farther, there is more coverage and less marking (López, 2017).

A total of 2,600 actions were observed. With respect to the defense actions of the player with the ball, the players developed 831 markings, 115 blockings, and 400 tacklings. In relation to the defense actions to the player without the ball, the players developed a total of 892 markings, 234 interceptions, and 128 help coverages.

All the game actions were recorded in official matches (two parts of 20 min at stopped clock) using a video camera, recording angle conversion lens (× 0.75): VCL-HGA07B and a Hama Gamma tripod Series. The camera was always placed in the background of the playing field, at a height of 4 m, guaranteeing an optimal view of all the game actions. Videos were transferred to a computer (Acer Aspire E15). Subsequently, data were recorded on a Microsoft Office Excel 2010 sheet and exported to the SPSS Inc., Released 2009 (PASW Statistics for Windows, Version 18.0, Chicago: SPSS Inc.).

### Reliability

With respect to the interobserver reliability, a research observer was trained to analyze decision-making and the execution of marking (to the player with and without the ball), blocking, tackling, interception, and help coverage. He was trained by a soccer expert (Level 2 by the Spanish Soccer Federation), who also had 4 years’ experience in observational methodology (researcher with experience in research projects).

As a preparatory stage to the observations, the expert met with the observer to clarify possible doubts about the observation instrument and the coding criteria of each dependent variable (DM and Ex) on the actions mentioned. Then, the observations were carried out. A total of 125 markings, 18 blockings, and 60 tacklings (defense actions of the player with the ball) and 134 markings, 35 interceptions, and 19 help coverages were analyzed, using a sample higher than 10% of the total (Tabachnick and Fidell, 2007). Interobserver reliability was calculated using the following formula: agreements/(agreements + disagreements) × 100 measure. Once this value was calculated, the Cohen kappa index was used. Values above 0.90 were obtained for all training sessions, surpassing the value of 0.81 from which adequate concordance is considered (Fleiss et al., 2003), thus achieving the necessary reliability for the subsequent coding of the dependent variables.

To guarantee the time reliability of the measurement, the same coding was developed at two different moments, with a time difference of 10 days. Cohen kappa values were found to be higher than 0.92, which reflected a reliable concordance.

### Statistical Analysis

The statistical software SPSS Inc., Released 2009 (PASW Statistics for Windows, Version 18.0, Chicago: SPSS Inc.) was used for data analysis and processing. Data normality was examined and confirmed by the Shapiro–Wilk test, which led to the use of parametric statistics. Descriptive statistics were calculated, obtaining the mean (M) and the standard deviation (SD) for all variables; to examine the possible differences between the two phases considered in the study, preintervention and intervention, a multivariate analysis of variance (MANOVA) of repeated measurements of a single group was carried out. The Bonferroni’s *post hoc* test was used to make multiple paired comparisons and identify the significant differences. Effect sizes were calculated using the partial eta-squared statistic (η*_*p*_*^2^). The effect size (ES) was classified as no effect (η*_*p*_*^2^ < 0.04), minimum effect (0.04 < η*_*p*_*^2^ < 0.25), moderate effect (0.25 < η*_*p*_*^2^ < 0.64), and strong effect (η*_*p*_*^2^ > 0.64) (Ferguson, 2009). The level of statistical significance was established at *p* ≤ 0.05, with a confidence interval for differences set at 95%.

## Results

The pairwise comparisons between the two phases of the study regarding the action are presented.

### Defending Actions to Player With the Ball

The analysis of the marking actions to the player with the ball revealed significantly higher values for the intervention phase compared to the preintervention phase in DM (*p* = 0.001) and Ex (*p* = 0.001). On the other hand, for blocking actions, significantly higher values were observed in favor of the intervention phase compared to the preintervention phase in DM (*p* = 0.015), which did not happen for the Ex variable (*p* = 0.270). Finally, there were no significant differences in any of the variables analyzed for the tackling actions (DM, *p* = 0.498; Ex, *p* = 0.471) (see Table 3).

**TABLE 3:** Descriptive analysis and comparison by pairs of DM and Ex of defense players to the attacker with the ball.

	**Var.**	**Pre (I)**			**Int (J)**			***p***	**η *_*p*_*^2^**	**ES**	**Differences 95% CI**	
		**M**	**SD**	**CV%**	**M**	**SD**	**CV%**				**LL**	**UL**
Marking	DM	0.325	0.140	0.431	0.632	0.046	0.073	**0.001**	0.870	Strong	0.403	0.555
	Ex	0.247	0.078	0.316	0.490	0.097	0.198	**0.001**	0.835	Strong	0.307	0.431
Blocking	DM	0.982	0.047	0.048	0.762	0.156	0.205	**0.015**	0.655	Strong	0.801	0.844
	Ex	0.619	0.441	0.712	0.377	0.182	0.483	0.270	0.197	Minimum	0.303	0.693
Tackling	DM	0.863	0.108	0.125	0.830	0.083	0.100	0.498	0.080	Minimum	0.777	0.917
	Ex	0.670	0.103	0.154	0.641	0.136	0.212	0.471	0.090	Minimum	0.554	0.758

*Var, variable; M, mean; SD. standard deviation; CV%, coefficient of variation; ES, effect size; DM, decision-making; Ex, execution; Pre, preintervention phase; Int, intervention phase; CI, confidence interval; LL, lower limit; UL, upper limit. Values in bold indicate “significant difference” (p < 0.05).*

### Defending Actions to Players Without the Ball

The analysis of marking actions to the player without the ball revealed significantly higher values for the intervention phase compared to the preintervention phase in DM (*p* = 0.039) and Ex (*p* = 0.046). In contrast, no significant differences were found for any of the variables analyzed in the interception actions (DM, *p* = 0.777; Ex, *p* = 0.336). Finally, for help-coverage actions, significantly higher values were observed in favor of the intervention phase compared to the preintervention phase in Ex (*p* = 0.014), which did not occur for the DM variable (*p* = 0.132) (see Table 4).

**TABLE 4:** Descriptive analysis and comparison by pairs of DM and Ex of defense players to the attacker without the ball.

	**Var.**	**Pre (I)**			**Int (J)**			***p***	**η *_*p*_*^2^**	**ES**	**Differences 95% CI**	
		**M**	**SD**	**CV%**	**M**	**SD**	**CV%**				**LL**	**UL**
Marking	DM	0.521	0.130	0.250	0.691	0.133	0.192	**0.039**	0.535	Moderate	0.514	0.699
	Ex	0.518	0.132	0.255	0.671	0.126	0.188	**0.046**	0.513	Moderate	0.501	0.689
Interception	DM	0.981	0.031	0.032	0.979	0.027	0.028	0.777	0.014	No effect	0.954	1.007
	Ex	0.824	0.135	0.164	0.884	0.090	0.102	0.336	0.154	Minimum	0.775	0.935
Help-coverage	DM	0.762	0.221	0.290	0.934	0.126	0.135	0.132	0.337	Moderate	0.733	0.963
	Ex	0.480	0.286	0.596	0.915	0.153	0.167	**0.014**	0.663	Strong	0.552	0.844

*Var, variable; M, mean; SD, standard deviation; CV%, coefficient of variation; ES, effect size; DM, decision-making; Ex, execution; Pre, preintervention phase; Int, intervention phase; CI, confidence interval; LL, lower limit; UL, upper limit. Values in bold indicate “significant difference” (p < 0.05).*

## Discussion

The aim of this study was to analyze the indirect effects of an intervention program, based on NLP (task design based on tactical principles of attack and numerical advantage of attacking team), in DM and Ex in defensive technical–tactical actions in U16 futsal. These variables were analyzed considering, on the one hand, the defender that marked the player with the ball (marking, blocking, and tackling) and, on the other hand, the defenders that marked the attacking players without the ball (marking, interception, and help coverage).

Generally, results revealed that, for defending actions to the players with the ball, players improved DM and Ex of marking actions and DM for blocking actions. In addition, in the defending actions to the player without the ball, players improved DM and Ex of marking actions and Ex for help-coverage actions. Any other variable revealed improvement after the training program.

In relation to the defensive actions to the attacking player with the ball, an improvement in the DM and Ex of *marking* was observed. It means that defensive players try to maintain the alignment with the goal according to the ball position. Thus, such results highlight that numerical unbalance help defensive players to focus their attention to informational variables that sustain their actions of marking, even in a context of high variability and uncertainty (Travassos et al., 2014b). Marking in futsal, for example, can be developed with an opponent attempting to pass the ball (perform under offensive pressure based on an attack player with the ball or not) and under variable task constraints [game rules, numerical balanced (i.e., 1vs2, 2vs3…) and space].

Regarding the improvement in the DM of the *blocking*, previous analysis of the spatial–temporal principles that shaped successful shoot interceptions revealed that defenders seek to maintain their position between the ball and the goal, not allowing a misalignment between the ball and the goal (Vilar et al., 2012). As with previous studies, variability in the attacking players’ relations with opponents and the ball is attributed to their constant explorative performances as they seek to break symmetry with the defending players in view of creating opportunities for scoring goals (Corrêa et al., 2012). However, the explorative behaviors of the attacking team take place under the constraints imposed by the behaviors of the defending team. As noted, the latter tries to maintain spatial–temporal relations with the former, whereas the former attempts to disrupt the *status quo* at opportune times, by advancing position in the field, reaching the free attacking player, and finding chances for goal-scoring possibilities (Travassos et al., 2014b). Thus, it can be argued that a large percentage of tasks worked, indirectly, on the content “obstruction of pass lines and shots.”

In line with the first argument, due to the fact that defense always played in numerical inferiority, on very few occasions, players were able to “tackle and try to regain the possession,” a very serious error in this type of situations (López, 2017). Therefore, the options for making successful tackling decrease considerably. Instead, it must be timed, obstructing passing lines to reverse this situation of superiority and turning it into an apparent equality. Previous studies such as that carried out by Travassos et al. (2014b) indicate that, when the defending team is in numerical inferiority, the distance between the defenders decreases and the defender–attacker distance increases. Therefore, how are players going to do tackling if the attackers are so far away?

In line with previous paragraphs, NPL advocates the use of open contexts impregned of variability and uncertainty but in which the manipulations guide players to become more proficient at perceiving environment cues and constant changes in game situations (Santos et al., 2016). This is an important way to facilitate the emergence of novel and functional solutions through adaptive movement patterns. In this sense, the use of more ecological training situations allows players to attune relevant sources of information based on information–movement coupling. Consequently, the manipulation of task constraints is extremely important to promote randomness in player’s actions. Moreover, this dynamical change develops exploratory behavior that encourages players to discover new action possibilities (Santos et al., 2016). In relation to *marking* (defensive actions of the attacking player without the ball), the numerical superiority in training tasks can help to improve players’ attunement to information through the reduction in the information that players need to pick up to perform.

Regarding the non-improvement in the *interception* actions, it can be pointed out that, in the training tasks, there is hardly any possibility that these will happen. Thus, there is no transfer to the real matches. As Travassos et al. (2014b) point out that, when players are in numerical inferiority, they get closer to each other and close the spaces trying to protect the goal. This means that defenders restricted space between themselves and, consequently, the occupied area in front of the goal (Sampaio et al., 2014), thereby restricting space for external kicks, diagonal or longitudinal passes to the free player (Travassos et al., 2011). In this regard, usually the defender near the ball approaches the attacker with the ball to avoid progression and the defender that is marking the attacker without ball tends to close crossing passing lines that disrupt defensive structure or shooting lines to the goal (Travassos et al., 2011). On the other hand, following the informational constrains that sustain successful passes vs. interceptions of passes, changes in emergent spatial–temporal interactions and the consequent patterns of coordination between teams are expected between game conditions (Vilar et al., 2014). In this sense, these constraints are likely promoting changes in the breadth of attention and in tactical behavior of players (Memmert and Roth, 2007). However, our results suggest that playing with one less defender (underload) might not impact greatly on the capacity of a defensive team to intercept the passes by players in the attacking team.

Referring to *help-coverage* actions, the fact of having worked in numerical inferiority has allowed a greater breadth of attention of defenders to protect the position of the defender that is marking the attacker with the ball. In line with that, Torrents et al. (2016) revealed that the use of numerical unbalance game contexts when compared to balance ones allows players to explore more individual and collective tactical/technical actions that support their success. It is likely that, in this study, even though the help-coverage actions do not make the appropriate decisions, this low skill of the attacking player in the dribbling action allows to achieve success in the Ex of the defensive action.

Summarizing, the development of training exercises with numerical disadvantage promotes strong couplings between players of the defending team, the ball position, and the goal and not with the attacking players, which demonstrates how the defenders prioritize protecting the goal against ball displacements more so than against movements of the attackers (Tenga et al., 2010). As observed in previous research (Travassos et al., 2012a), it seems that the defending team adopted a zone defense with the focus of maintaining all players between the ball and the goal, resulting in the defending players trying to move in synchrony with the ball and so maintain balance with the attacking team. This behavioral change shows a tactical adaptation of the defending team under changing game conditions to constrain space and time of the attacking team. This tactical behavior assists the defending players in obtaining extra time for positioning themselves on the field to maintain spatial–temporal pressure on the attacking team to try and close their shooting and, especially, their passing lines (Travassos et al., 2012a).

The current study had several strengths. First, instruments with sound reliability and validity were utilized to collect the data. Second, the novelty of this study is that there are no researches that have sought to investigate the effects of intervention program in defensive actions. Despite the aforementioned strengths, the study results should be treated with some caution due to the utilization of a small sample, which limits the capacity to extrapolate the results. Future studies should be developed with a larger sample (that could minimize the effects of other factors on tactical development, especially when researching amateur players) and with teams of different age categories, gender, and levels of expertise to improve the understanding of this issue. On the other hand, the program has been carried out in natural context, where some contextual variables are difficult to control. In this sense, players’ decision-making and execution could be affected by the contextual variables as outcome or current score (potentially affecting motivation and playing behaviors) (Lupo and Tessitore, 2016). In addition, due to the level of players (average skill level of sport expertise), we can hypothesize that a longer intervention program and more matches to verify the players’ behaviors could be provided to make more solid results. Future research in this line is necessary to provide scientific knowledge and help coaches to improve their intervention programs and better control the learning process of players.

## Conclusion

This study has shown evidence that the NLP approach is an appropriate theoretical framework to enhance acquisition of defensive tactical behavior in futsal. Our results come to reinforce that the use of numerical unbalance in defense promotes not only acute but also chronic effects in players’ tactical behavior. Additionally, the variability of practice led to searching answers to different problems and always with actions. In this sense, despite of all the efforts of the coach in this program destined to unify the understanding and improvement of the attack, not keeping in mind the defensive phase, players improved their defensive tactical capability to perform (in marking, blocking, and help coverage). However, not all actions were improved. Specifically, marking action improvements are related with tasks with numerical advantage of the offensive team. However, if coaches want to improve interception actions, offensive numerical superiority tasks are not the best option. This study demonstrated the need for coaches to identify the development of defensive (or offensive) actions in small-sided games with duality of purposes. Therefore, these results provide practitioners with important insights on how they can better organize their training sessions and design representative tasks to optimally develop technical–tactical training processes based on the phases of futsal game.

## Data Availability Statement

All datasets generated for this study are included in the article/supplementary material, further inquiries can be directed to the corresponding author.

## Ethics Statement

The studies involving human participants were reviewed and approved by the Ethics Research Committee of a Spanish University. Written informed consent to participate in this study was provided by the participants’ legal guardian/next of kin.

## Author Contributions

DP developed data collection and wrote the first draft of the manuscript. AP, BT, and AM contributed to design of the study, data analysis and interpretation, and wrote sections of the manuscript. All authors contributed to manuscript revision, read and approved the submitted manuscript.

## Conflict of Interest

The authors declare that the research was conducted in the absence of any commercial or financial relationships that could be construed as a potential conflict of interest.
